# EHMTI-0387. Characterization of peripheral and central sensitization in patients undergoing occipital nerve stimulator implant for intractable migraine

**DOI:** 10.1186/1129-2377-15-S1-M8

**Published:** 2014-09-18

**Authors:** V Mehta, A Bahra, L Casey, A Alamgir, R VanGroningen, T Wodehouse

**Affiliations:** 1 Pain & Anesthesia Research Centre St Bartholomew's Hospital, Bart's Health NHS Trust, London, UK; 2 Department of Neurology Whipps Cross Hospital, Bart's Health NHS Trust, London, UK; 3 Department of Neurosurgery Royal London Hospital, Bart's Health NHS Trust, London, UK

## Introduction

Trigemino-spinal sensitization and impaired descending inhibitory control (DNIC) has been reported in chronic migraine.1 Further mechanism behind occipital nerve stimulation (ONS) remains speculative.

## Aims

This observational study characterises peripheral and central sensitization in patients undergoing ONS for intractable chronic migraine

## Methods

Quantitative Sensory testing (QST) measurements were carried out in patients before and after ONS (n=6).

1. Measurement of pressure pain thresholds (PPT): Computer-controlled pressure algometer (SomedicAB, Sweden, diameter contact tip 10mm; standardised speed 0.3kg/s) measured PPTs at standardized three points (temple, cheek bone and cervical).

2. DNIC: DNIC response was measured using PPT on cheekbone with an inflated cuff insitu on one arm.

## Results

Patients with chronic migraine demonstrated loss of DNIC, (PPTs 56.0 KPa vs 46.2kPA cuff inflated). A “normal’ DNIC response was observed two weeks following ONS (58.4 kPA vs 100.4kPA cuff inflated) continuing positively over next three months. In contrast the PPTs remained same before and after the ONS.

## Conclusion

This case series reports sustained reversal of the loss of DNIC response in patients undergoing ONS for intractable headache whilst having no effect on peripheral pain thresholds.

No conflict of interest.
